# A Case of Unresectable Hepatocellular Carcinoma Treated with Spacer Placement Surgery with Bioabsorbable Spacer and Subsequent Proton Beam Therapy

**DOI:** 10.70352/scrj.cr.25-0026

**Published:** 2025-04-22

**Authors:** Toru Takahashi, Shohei Komatsu, Yusuke Demizu, Keisuke Arai, Nobuaki Ishihara, Akihiro Fujisawa, Hidetoshi Gon, Hirochika Toyama, Sunao Tokumaru, Takumi Fukumoto

**Affiliations:** 1 Department of Surgery, Division of Hepato-Biliary-Pancreatic Surgery, Kobe University Graduate School of Medicine, Kobe, Hyogo, Japan; 2 Department of Radiation Oncology, Hyogo Ion Beam Medical Center Kobe Proton Center, Kobe, Hyogo, Japan

**Keywords:** hepatocellular carcinoma, proton beam therapy, spacer placement surgery, case report

## Abstract

**INTRODUCTION:**

Hepatocellular carcinoma (HCC) often requires repeated therapy and poses challenges in treatment selection, particularly in patients with impaired liver function. Although hepatic resection, radiofrequency ablation, and liver transplantation are standard local curative therapies, the position of radiotherapy, including proton beam therapy (PBT), remains relatively underexplored. Herein, we report an illustrative case of unresectable HCC treated with spacer placement surgery using a bioabsorbable spacer, followed by PBT.

**CASE PRESENTATION:**

We report the case of a 77-year-old male patient diagnosed with a 6 cm HCC in segment 8, accompanied by impaired liver function, precluding hepatic resection. PBT was planned; however, because of the proximity of the gastrointestinal tract to the tumor, spacer placement was deemed necessary, and a bioabsorbable polyglycolic acid spacer was placed, followed by PBT. Owing to the sufficient space provided by the spacer, curative doses of PBT could be delivered to the tumor, and the patient survived for 26 months after spacer placement surgery without any sign of recurrence.

**CONCLUSIONS:**

Bioabsorbable spacer placement surgery and subsequent PBT are feasible and promising treatment options for unresectable HCC with impaired liver function.

## INTRODUCTION

Hepatocellular carcinoma (HCC) is a cirrhotic liver tumor that may require repeated therapy. Hepatic resection, liver transplantation, radiofrequency ablation, and transcatheter arterial chemoembolization are standard treatments for localized HCC. Treatments are selected based on the patient’s liver function, tumor size, and tumor number. If these treatments are not suitable or there is no consensus, radiotherapy can be an alternative treatment option.^1,2)^ Stereotactic radiation therapy for HCC has enabled the delivery of curative doses owing to advances in irradiation techniques, and has achieved a high local control rate, especially for small lesions.^2)^ Particle therapy, including proton beam therapy (PBT) and carbon ion radiotherapy, has an excellent dose concentration and can safely deliver high doses to tumors with favorable local control for large lesions. Recent studies have shown that the 5-year local control rate of particle therapy is as high as 80%–90% and is efficacious for local control.^3,4)^ These findings suggest that the indications of particle therapy as a curative treatment for HCC are likely to expand.^2,5)^

The higher dose distribution of particle therapy needs to be carefully handled, considering the uncertainties and tissue changes that potentially expose organs, especially the gastrointestinal tract, to doses that exceed their tolerances. This may result in significant side effects, such as intestinal adhesions, gastrointestinal bleeding, or intestinal perforations, when escalating the dose to the target.^6)^ As gastroenteric toxicity following particle therapy can be a negative prognostic factor, radiation dose reduction may be required, potentially leading to local recurrence.^7)^ To avoid these problems, space-making strategies such as space-making particle therapy (SMPT) have been suggested, in which medical materials are used to separate the tumor from the adjacent gastrointestinal tract to safely increase the dose delivered to the target tumor.^8)^ Here, we report a case of HCC that was difficult to cure and was treated with SMPT using bioabsorbable spacers.

## CASE REPORT

**Fig. 1:**
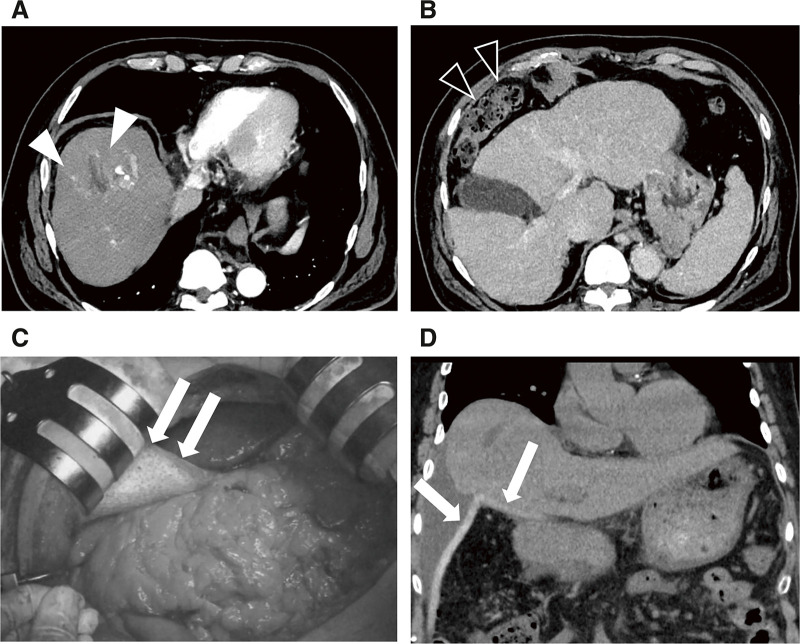
Abdominal computed tomography before and after the spacer placement surgery and intraoperative images. (**A**) Abdominal computed tomography revealing a 6 cm tumor located at segment 8 (white arrowheads). (**B**) The colon is located on the ventral side of the liver (black arrowheads). (**C**) A polyglycolic acid spacer (white arrows) is placed caudally to the liver. (**D**) Coronal slice computed tomography scan of the abdomen after the spacer placement surgery showing that the polyglycolic acid spacer and omentum prevented the colon from covering the ventral side of the liver (white arrows).

A 77-year-old male patient followed for steatotic hepatitis was referred to our department with a 6-cm hepatic tumor in segment 8. His medical history included fatty hepatitis and hypertension. Laboratory data showed serum albumin levels of 3.8 g/dL (normal value, 4.1–5.1 g/dL), total bilirubin levels of 1.2 mg/dL (normal value, <0.2 g/dL), platelet count of 9.5 × 10^4^/uL (normal value, 15.8–34.8 × 10^4^/μL), and a Child–Pugh score of 6. The serum levels of alpha-fetoprotein and protein induced by vitamin K absence or antagonist II were 4 ng/mL (normal value, <10 ng/mL) and 92 mAU/mL (normal value, <40 mAU/mL), respectively. The indocyanine green retention rate at 15 minutes was 24.9% (normal range, <10%), and asialoscintigraphy also showed decreased liver function with HH15 of 0.77 (normal value, 0.5–0.6) and LHL15 of 0.86 (normal value, 0.91–0.96). The albumin–bilirubin (ALBI) score was −2.36 and modified ALBI grade was 2a. Abdominal computed tomography (CT) revealed a 6 cm tumor in segment 8, in which arterial enhancement was observed with rapid clearance during the delayed phase, and the patient was diagnosed with HCC. No apparent intra- or extrahepatic metastases were detected. The patient had Chilaiditi syndrome with the colon on the ventral side of the liver (**Figs. 1A** and **1B**).

Owing to significant hepatic decompensation, hepatic resection was deemed unsuitable, and PBT was planned with curative intent. The tumor was located in segment 8 of the liver; thus, PBT was planned for delivery from the ventral side of the patient. However, the transverse colon was located on the ventral side of the tumor, which preluded irradiation from the ventral side. Accordingly, spacer placement surgery was deemed necessary for safe and curative PBT, and spacer placement surgery with a bioabsorbable polyglycolic acid (PGA) spacer was planned, although it was not the standard clinical practice.

The abdomen was opened through a midline incision and spacer placement was performed after confirming the absence of distant intra-abdominal metastases. One 5-mm thick PGA spacer was placed caudally to the liver to prevent the transverse colon from covering the ventral side of the liver. The spacer was fixed to the retroperitoneum and ventral peritoneum of the liver using absorbable sutures to prevent displacement (**Fig. 1C**). The greater omentum was placed between the PGA spacer and the transverse colon to prevent direct contact between the two.

The postoperative CT confirmed that the PGA spacer prevented the colon from covering the ventral side of the tumor, and sufficient space was obtained between the tumor and the gastrointestinal tract (**Fig. 1D**). Although the patient was discharged on postoperative day 7 without any complications, he soon afterwards required hospitalization and ascites control. After controlling the ascites with diuretics, PBT was initiated on the 22nd postoperative day with a total of 66 Gy (relative biological effectiveness) in 10 fractions, and the patient completed PBT on the 35th postoperative day.

**Fig. 2:**
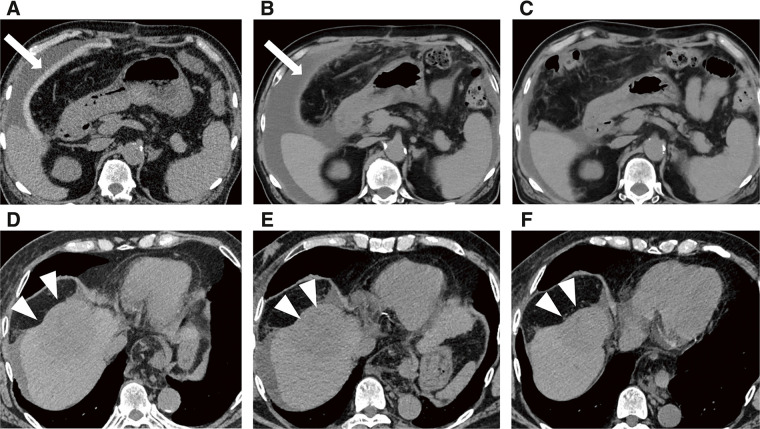
Changes in the abdominal computed tomography of the polyglycolic acid spacer and the tumor after the treatment. Abdominal computed tomography taken at (**A**) 1 month, (**B**) 2 months, and (**C**) 3 months after proton therapy showing that the polyglycolic acid spacer was gradually absorbed and disappeared (white arrows). Abdominal computed tomography taken (**D**) 3 months, (**E**) 12 months, and (**F**) 26 months after proton beam therapy, revealing no apparent sign of recurrences (white arrowheads).

**Fig. 3:**
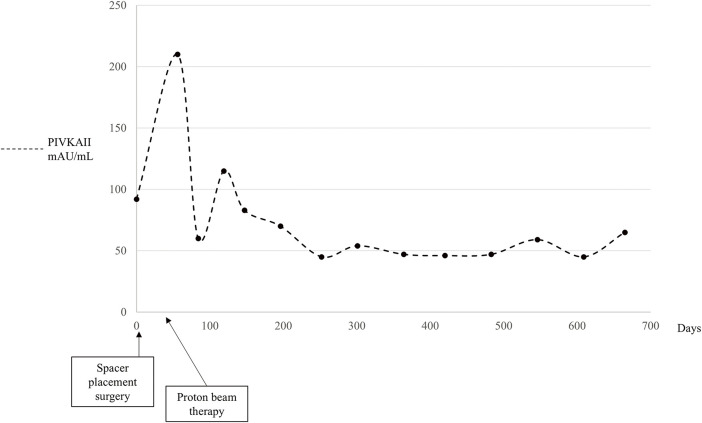
Transition of the serum levels of protein induced by vitamin K absence or antagonist II during the treatment course. The serum protein induced by vitamin K absence or antagonist II levels remained almost normal over the treatment course. PIVKAII, protein induced by vitamin K absence or antagonist II

**Fig. 4:**
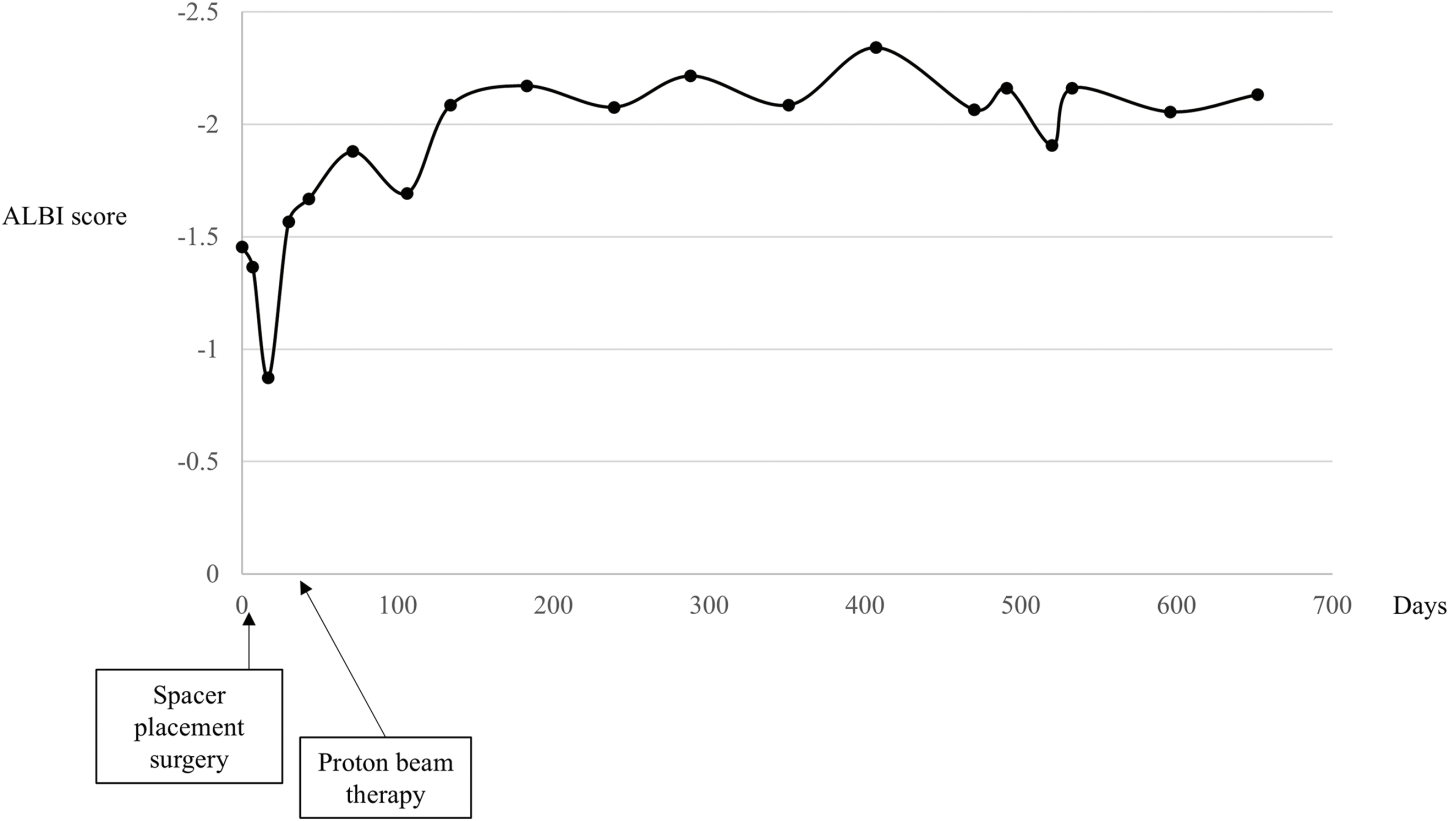
Transition of the albumin–bilirubin score during the treatment course. The ALBI score decreased significantly immediately after spacer placement surgery, thereafter, the score gradually improved and remained stable over the treatment course. ALBI, albumin–bilirubin

The patient was followed up without any severe complications after the treatments, and the PGA spacer was gradually absorbed and disappeared on CT scans 3 months after PBT (**Figs. 2A**–**2C**). There had been no apparent signs of recurrence by the time of the follow-up CT in November 2024, and the serum protein induced by vitamin K absence or antagonist II levels remained almost normal 26 months after PBT (**Figs. 2D**–**2F** and **Fig. 3**). The ALBI score did not worsen after SMPT, and the transition of the ALBI score is shown in **Fig. 4**. Regarding late radiation toxicities, a grade 2 rib fracture, grade 1 dermatitis, grade 1 pneumonitis, and grade 1 soft tissue fibrosis were observed; however, no treatment was needed.

## DISCUSSION

Although the position of particle therapy in the treatment algorithm of HCC remains unknown, several reports have shown the effectiveness of particle therapy for HCC in terms of both local control and overall survival.^9,10)^ Favorable local control with less toxicity is a significant advantage of particle therapy; thus, there is a great demand for it in the treatment of HCC.

SMPT is an upcoming novel concept, the strategy of which lies in the attempt to shield the organs that have a low radiation tolerance from the radiation field by spacer placement surgery.^11)^ The effectiveness and potential to expand the therapeutic indication of SMPT for several malignant tumors have been demonstrated by a rapid increase in the number of publications.^11–13)^ SMPT using expanded polytetrafluoroethylene spacers for HCC has already been reported, and clinical evidence has been established.^11,13)^ However, because the expanded polytetrafluoroethylene spacer is non-absorbable, some significant adverse events such as gastrointestinal perforation have occurred during long-term follow-up.^8,14)^ Therefore, owing to the development and clinical availability of PGA spacers, we use PGA spacers for SMPT in all cases after approval of clinical application. The advantages of the PGA spacer are that it is an absorbent material and its thickness can be customized according to tumor shape or anatomic difficulty.^15)^ Several previous reports that focused on the other malignancies demonstrated the effectiveness of SMPT with a PGA spacer.^16,17)^ In this case, in addition to impaired liver function, Chilaiditi syndrome was noted on preoperative CT, and spacer placement surgery was necessary for safe and curative PBT. Traction and fixation of the caudal side of the omentum alone were insufficient, as there was a risk that the transverse colon would be located ventral to the liver. Therefore, spacer placement surgery was essential to prevent the elevation of the colon toward the diaphragm. Postoperative CT showed that the gastrointestinal tract was completely excluded from the radiation field, and curative doses were delivered to the tumor. The patient required postoperative ascites control because of decreased liver function, although hepatic resection was not performed. If hepatic resection had been performed, liver failure was a significant possibility; thus, SMPT with a PGA spacer proved to be a safer and more suitable treatment option for this complex case. The PGA spacer completely absorbed 3 months postoperatively, and no late adverse events were noted. This case report uniquely highlights the application of a PGA spacer specifically for HCC. To the best of our knowledge, this is the first report of a radical cure of HCC achieved using SMPT with a PGA spacer, representing a significant advancement over previous treatment.

Aside from Chilaiditi syndrome, spacer placement surgery for HCC is indicated for tumors located in areas close to the gastrointestinal tract, such as the inferior surface of the lateral segment or hepatic segments 5 and 6. Since spacer placement surgery is a preparatory procedure for particle therapy, it is essential to maintain a minimally invasive approach and avoid touching the tumor as much as possible to prevent the risk of tumor manipulation. Additionally, efforts should be made to keep the incision as small as possible, reduce bleeding, and shorten the operative time.

Particle therapy was approved by public health insurance for large HCC (≥4 cm in diameter) cases in April 2022 in Japan, and expanded indications are expected. When comparing the initial treatment for patients with a single HCC without vascular invasion between hepatic resection and particle therapy, the prognosis was significantly better for hepatic resection than for particle therapy for patients with preserved liver function.^18)^ However, prognosis was equal for patients with impaired liver function with either hepatic resection or particle therapy, suggesting the significant potential of particle therapy for patients with a single HCC who cannot undergo hepatic resection due to impaired liver function, as in the present case.^18)^

Particle therapy contributes to expand the curative local treatment options, especially in cases that are difficult to treat with surgery, and SMPT may further expand treatment options.

## CONCLUSION

Bioabsorbable spacer placement surgery and subsequent PBT are feasible and promising treatment options for unresectable HCC with impaired liver function.

## DECLARATIONS

### Funding

No funding was received.

### Authors’ contributions

TT and SK conceptualized the case report.

TT wrote the manuscript and performed additional data analysis.

SK, YD, KA, NI, AF, HG, HT, ST, and TF were involved in the treatment and follow-up in this case.

SK, YD, KA, NI, AF, HG, HT, and ST critically revised the manuscript and provided valuable feedback.

TF provided supervision and approved the final manuscript for publication.

TT and SK confirmed the authenticity of all the raw data.

All authors have read and approved the final version of the manuscript.

### Availability of data and materials

The data generated in the present study may be requested from the corresponding author.

### Ethics approval and consent to participate

This case report describes an individualized treatment based on the patient’s condition and clinical evidence. Therefore, it is classified as an exempt study and does not require formal ethical approval. Written informed consent was obtained, and the study adhered to ethical standards, respecting patient privacy.

### Consent for publication

Written informed consent for publication of individual data and any accompanying images were obtained from the patient.

### Competing interests

The authors declare that they have no competing interests.
